# Biomimetic Scaffolds for Regeneration of Temporomandibular Joint Disc: A Narrative Review

**DOI:** 10.30476/dentjods.2023.97625.2024

**Published:** 2024-06-01

**Authors:** Hojat Rezazadeh, Nazafarin Samiraninezhad, Mostafa Rezaee

**Affiliations:** 1 School of Dentistry, Shiraz University of Medical Sciences, Shiraz, Iran; 2 Dept. of Oral and Maxillofacial Medicine, School of Dentistry, Shiraz University of Medical Sciences, Shiraz, Iran

**Keywords:** Temporomandibular Joint Disc, Regeneration, Tissue Scaffolds, Hydrogels, Decellularized Extracellular Matrix

## Abstract

Defects and dysfunctions of temporomandibular joint (TMJ) disc are responsible for the majority of TMJ diseases. Current treatments in this matter are usually short-term and only palliative, thus an alternative treatment that offers long-lasting repair is in great demand. In recent years great attempts have been made to prepare an ideal scaffold, which best resembles the native TMJ disc in characteristics such as mechanical, physical and biological properties. This narrative review focuses on developments of the recent ten years in fabrication of scaffolds using decellularized tissues, natural and synthetic biomaterials for regeneration of TMJ disc and compared their properties. PubMed and Google Scholar databases were searched using the following keywords (“TMJ” OR “temporomandibular joint” OR “TMD” OR “temporomandibular disease”) AND (“scaffold” OR “hydrogels”). Randomized controlled trials, randomized clinical trials, case-controls, case reports, and animal studies were included. Comments, systematic reviews, meta-analyses, and non-English papers were excluded. The study concluded that hybrid scaffolds have exhibited favorable cell attachment and proliferation. Synthetic scaffolds have shown promise in providing better control over structural properties; however, additional processes are often required to provide biomimetic cell signaling. While there is still much to learn about the ideal scaffold for TMJ disc regeneration, both natural and synthetic scaffolds have shown promise in achieving the functional, structural, biological, and mechanical properties of a native TMJ disc.

## Introduction

**Figure 1:**
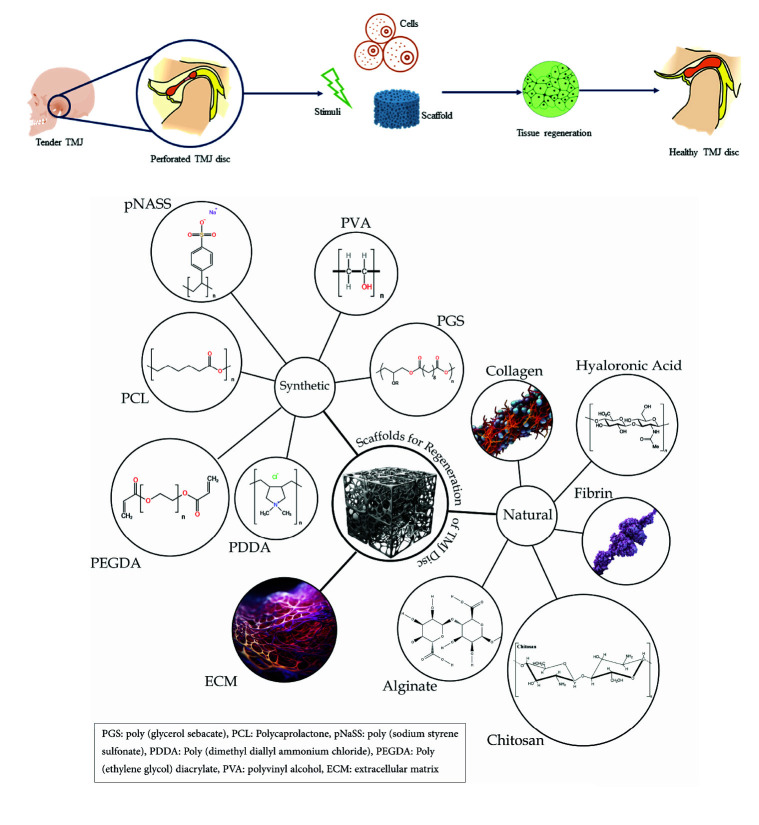
Tissue engineering for TMJ disc regeneration

Temporomandibular joint disease (TMD) excruciatingly reduces the comfort and quality of life of many people worldwide [ 1 ]. TMD occurs as a result of dysfunctions or defects in the temporomandibular joint (TMJ) disc such as disc displacement which is believed to increase the possibility of perforation [ 2 ]. The importance of TMJ disc is that it acts as a stress absorber and a distributor of stress [ 3 ]. Disc may not recover from a perforation due to avascular nature, which eventually leads to impaired function [ 2 ]. Several surgical options are suggested to relieve the pain such as discectomy; however, they are only anodynes, which usually require revision and come with significant post-surgical complications [ 4 - 5 ]. Thus, tissue engineering has been studied as an alternative option for a rather long time and attempts have been made to prepare a scaffold that best resembles the features of native TMJ disc (Figure 1). 

The native TMJ disc is a fibrocartilage, which mostly consists of water, as well as, cells and extracellular matrix (ECM) [ 6 ]. Cellular and extracellular elements of TMJ disc have shown region-dependent distribution [ 5 - 6 ], which is considered to be responsible for viscoelastic and anisotropic tensile properties [ 7 - 8 ]. Approximately 70% of cellular element is composed of fibroblast-like cells and the rest is composed of chondrocyte-like cells.

The former is found all over the tissue, while the latter is mostly found in the intermediate area [ 9 ]. The major part of ECM is made up of collagen fibers (mostly type 1) which mainly have circumferential orientation in the peripheral area and anteroposterior orientation in the intermediate area, and the rest is composed of elastic fibers and glycosaminoglycans (GAG). GAGs are mainly localized in the intermediate area [ 5 ]. 

Aside from diverse composition and fiber orientation in different parts of the disc, mechanical properties (to withstand compressive, tensile and shear forces), biological properties (suitable for cell proliferation and differentiation), and physical properties (to provide mass transport) are all the challenges in regeneration of TMJ disc [ 6 , 9 - 10 ].

In this narrative review we aimed to study materials that have already been fabricated and examined (whether *in vitro* or *in vivo*) to prepare an inhomogeneous tissue capable of replacing TMJ disc. 

## Search Strategy

### Methods and search strategy

PubMed and Google Scholar databases were analyzed with the following keywords: (“TMJ” OR “temporomandibular joint” OR “TMD” OR “temporomandibular disease”) AND (“scaffold” OR “hydrogels”) 

### Inclusion and exclusion criteria

Randomized controlled trials, randomized clinical trials, case-controls, case reports, animal studies from the last 10 years (since 2013) were included. Comments, systematic review and meta-analyses and papers that were not written in English were excluded. 

## Discussion

Scaffolds are defined as support structures for tissue formation [ 11 ]. Natural biomaterials, synthetic biomaterials, and decellularized tissues have widely been used as scaffolds in TMJ disc regeneration [ 12 - 13 ]. Different materials and modifications have been investigated to achieve the best scaffold that can mimic functional, structural, biological, and mechanical properties of a native TMJ disc. Table 1 presents the included *in vitro* studies; Table 2 presents the included *in vivo* studies, and the scaffolds that have been used with their results.

**Table 1:** Scaffolds used for TMJ disc regeneration in *in vitro* studies

Article	Scaffold material	Fabrication technique	Reinforcement material	Seeded cells	Results
Hagandora *et al*. [ 40 ], 2013	PGS	NR	-	Fibro-chondrocyte	Higher cellular content, and EMC production with an increase in fibro-chondrocytes seeding density, random distribution of collagen and GAG
Wu *et al*. [ 24 ], 2014	Chitosan/fibrin	Freeze-drying	-	TMJ derived MSCs	Increased cell seeding efficiency and biocompatibility, increased production of GAG and collagen type 1, increased cell distribution
Juran *et al*. [ 10 ], 2015	porcine TMJ disc-derived decellularized ECM	Decellularization modified by laser micropatterning	-	Human umbilical cord MSC	Increased compressive modulus, decreased hydraulic permeability coefficient, improved cell adhesion, proliferation, cellular migration and metabolic activity
Ronald *et al*. [ 4 ], 2016	pNaSS and PDDA	LBL	Cationic TiO2 nanoparticles	Fibro chondrocyte	Cytocompatible and cell adherent, promoting cellular growth, Fibro chondrocyte proliferation, increased protein expression, increased collagen type 1, decorin, aggrecan and collagen type 2
Legemate *et al*. [ 5 ], 2016	PCL	LBL	CTGF and TGFβ3	Human BMSC	Sustained release of CTGF and TGFβ3 up to 42 days, formation of heterogeneous fibrocartilaginous tissues, higher collagen contents in the intermediate zone and the anteroposterior band, increased GAG contents, coefficient of viscosity and compressive modulus and tensile modulus nearly same as that of native discs
Tarafder *et al*. [ 30 ], 2016	PCL	LBL	BMP-2, CTGF and TGFβ3	Human BMSC	Spatial control of growth factor delivery, sustained re-lease of growth factor up to 42 days, formation of a c-ollagen-rich fibrous matrix, multi-lineage differentiation of BMSCs, region-variant tissue phenotype, imp-roved healing
Francisco *et al*. [ 6 ], 2017	PCL and PEGDA	PCL: LBL	-	-	Temperature of PCL preparation and PEGDA concentration influence mechanical properties
		PEGDA: Photopolymerization			
Bousnaki *et al*. [ 21 ], 2018	Chitosan/Alginate	Crosslinking	-	Dental pulp stem cells	Cell viability and proliferation up to 14 days, uneven distribution of cells, fibro-chondrogenic differentiation, expression of almost all late chondrogenic differentiation markers, formation of collagen type 1 and no expression of collagen type 2, cell aggregate formation with ECM deposition after 4 weeks, deposition of aggrecan
Liang *et al*. [ 1 ], 2020	TMJ disc-derived decellularized ECM	Modified decellularization	-	-	95% reduction in DNA content, a significant enrichment in collagen content and a dramatic reduction in GAG content, randomly oriented and dense fiber morphology and a porous structure, decreased viscosity with increase of shear rate, increased storage modules with increased temperature, increased compressive strength with increased concentration of ECM, biocompatible, no sign of ulceration or pus in mice, minor inflammation after implantation, volume reduction from 30 min until 7 days, numerous cells migrated into the hydrogel and even reached the central layer of the hydrogel on day 7, angiogenesis on the surface but not inside the hydrogel 7 days after implantation
Moura *et al*. [ 26 ], 2020	1. PCL+ PEGDA core	PCL: Fused deposition modeling	-	-	Decreased porosity and increased compressive modulus with increased temperature, decreasing the filament size (300 to 200 µm) decreases the compressive modulus, scaffolds with 200 µm filaments have closer modulus to the native disc, presence of PEGDA hydrogel results in better mechanical properties
	2.PCL+PEGDA shell	PEGDA: Photopolymerization			
Jiang *et al*. [ 27 ], 2021	PVA hydrogel	Crosslinking	3D-printed PCL implants	Fibroblast and chondrocyte	Mechanical strength similar to that of natural disc, capable of absorbing the destructive energy during loading, lower creep, lower maximum tensile stress than native TMJ however it was enough to tolerate normal functional movements, low friction, smooth surface, better fatigue resistance, better viscoelasticity, better hydrophilicity, non-adhesive nature, cytocompatible
Wang *et al*. [ 18 ], 2021	HA/Collagen type 1 blend hydrogel	Crosslinking	BCP	rabbit BMSC and chondrocyte	Proliferation and osteogenic differentiation and chondrogenic specific matrix secretion, degradable, higher GAG/DNA ration and chondrogenic phenotype genes in scaffold with chondrocyte, higher COL IA2 gene expression in scaffold with BMSC
Gan *et al*. [ 46 ], 2022	PCL/ PLA	Electrospinning and LBL	Carbon nanotubes	Rat BMSC	Optimal cell proliferation, aggrecan and collagen type 1 expression, biocompatibility, higher compressive stress and relaxation modulus

PGS: poly (glycerol sebacate), PCL: Polycaprolactone, pNaSS: poly (sodium styrene sulfonate), PDDA: Poly (dimethyl diallyl ammonium chloride), PEGDA: Poly (ethylene glycol) diacrylate, PVA: polyvinyl alcohol, PLA: Polylactide, HA: hyaluronic acid, ECM: extracellular matrix, LBL: layer-by-layer Nano assembly, BMP-2: bone morphogenetic protein 2, CTGF: connective tissue growth factor, TGF: transforming growth factor, BCP: biphasic calcium phosphate, BMSC: bone marrow mesenchymal stem cells, MSC: mesenchymal stem cells, NR: not reported, GAG: glycosaminoglycan

**Table 2:** Scaffolds used for TMJ disc regeneration in *in vivo* studies

Article	Scaffold material	Fabrication technique	Reinforcement material	Seeded cells	Results
Ahtiainen *et al*. [ 47 ], 2013	PLA	NR	TGFβ1	Adipose stem cell	Increased production of aggrecan and collagen type 1, complete healing after 6 months
Wu *et al*. [ 24 ], 2014	Chitosan/fibrin	Freeze-drying	-	TMJ derived MSCs	Increased cell seeding efficiency and biocompatibility, increased production of GAG and collagen type 1, increased cell distribution
Kobayashi *et al*. [ 2 ], 2015	Collagen	Crosslinking	-	Rabbit	Complete repair after 2 weeks with addition of stem cells
				BMSC	
Tarafder *et al*. [ 30 ], 2016	PCL	LBL	BMP-2, CTGF and TGFβ3	Human BMSC	region-variant tissue phenotype, improved healing
Liang *et al*. [ 1 ], 2020	TMJ disc-derived decellularized ECM	modified decellularization	-	-	no sign of ulceration or pus in mice, minor inflammation after implantation, volume reduction from 30 min until 7 days, numerous cells migrated into the hydrogel and even reached the central layer of the hydrogel on day 7, angiogenesis on the surface but not inside the hydrogel 7 days after implantation
Jiang *et al*. [ 27 ], 2021	PVA hydrogel	crosslinking	3D-printed PCL implants	Fibroblast and chondrocyte	caused no infection, able to maintain joint stability and protect condylar cartilage and bone from damage
Wang *et al*. [ 18 ], 2021	HA/Collagen type 1 blend hydrogel	crosslinking	BCP	rabbit BMSC and chondrocyte	regeneration of fibrocartilage and subchondral bone, smooth and well-integrated tissue after 6 weeks of implantation
Gan *et al*. [ 46 ], 2022	PCL/ PLA	Electrospinning and LBL	carbon nanotubes	Rat BMSC	more and denser collagen in vivo, fibro-chondrogenic capability

PGS: poly (glycerol sebacate), PCL: Polycaprolactone, pNaSS: poly (sodium styrene sulfonate), PDDA: Poly (dimethyl diallyl ammonium chloride), PEGDA: Poly (ethylene glycol) diacrylate, PVA: polyvinyl alcohol, PLA¬¬: polylactide, HA: hyaluronic acid, ECM: extracellular matrix, LBL: layer-by-layer Nano assembly, BMP-2: bone morphogenetic protein 2, CTGF: connective tissue growth factor, TGF: transforming growth factor, BCP: biphasic calcium phosphate, BMSC: bone marrow mesenchymal stem cells, MSC: mesenchymal stem cells, NR: not reported, GAG: glycosaminoglycan

### Natural Biomaterials

Despite the emergence of synthetic scaffolds, application of natural scaffolds has not obsoleted yet. This is mostly due to the hydrophobicity of synthetic scaffolds, which is believed to restrict cell attachment, a process that fundamentally affects cell signaling, migration and proliferation [ 14 - 5 ]. Protein-based scaffolds such as fibrin and collagen provide binding site for cell adhesion, while polysaccharide-based scaffolds such as alginate and chitosan require modification [ 16 ].

### Collagen

Collagen as a natural, biodegradable, and biocompatible material with high tensile strength has attracted a lot of attention in tissue engineering and been used in several forms including sponges [ 17 ]. Collagen sponge that is seeded with bone marrow stem cells is believed to completely repair a perforation of 1.6mm diameter in the center of the disc after 2 weeks [ 2 ]. A perforation in this size did not seem to be able to repair completely even after 2 months without either the scaffold or stem cells [ 2 ]. 

### Hyaluronic acid

Hyaluronic acid (HA) is one of the components of ECM whose scaffolds are famous for hydrophilicity and capability of chemical modification [ 16 ]. Addition of Thiolated hyaluronic acid polymer to collagen to fabricate an injectable hydrogel (HA-Collagen1) diminishes the degradation time and shrinkage of collagen hydrogel; however, it decreases the storage modulus and loss modulus, which means that the ability of the scaffold to either store energy elastically or dissipate stress decreases [ 18 ]. It also illustrates favorable compressive modulus, cell spreading, cell adhesion, and cell proliferation [ 18 ]. Biphasic calcium phosphate can be added to HA-Collagen1 to mimic the bone layer, while HA-Coll-agen1 mimics cartilage layer [ 18 ]. The combination still maintains its biocompatibility [ 18 ]. While comparing seeding either bone marrow stem cells or chondrocytes in HA-Collagen1-biphasic calcium phosphate hydrogel, the production of GAG is higher with the latter; however, production of aggrecan is higher with the former [ 18 ].

HA-Collagen1-biphasic calcium phosphate hydrogel seeded with both bone marrow stem cells and chondrocytes fully repairs a defect with 2mm diameter and 3 mm depth after 6 weeks. The regenerated tissue integrates well with the adjacent native tissue and have a homogenous distribution of collagen type 1 and 2 and same cell pattern as the native disc (parallel fibroblast-like cell in the superficial zone and columnar spherical cells in the deep zone). Absence of the cells results in an uneven reparative tissue, without collagen type 2 [ 18 ].

### Chitosan and Alginate

Chitosan and Alginate are natural, biocompatible, and biodegradable polymers [ 19 - 20 ]. Considering hydrophilicity of alginate and bioactivity of chitosan, a hybrid scaffold of these two polymers seeded with stem cells exhibits favorable cell attachment, proliferation, and chondrogenic differentiation [ 21 ]. Besides, collagen type 2, unlike collagen type 1, is not expressed at all. [ 21 ]. A noteworthy point is that addition of glutaraldehyde while preparing the chitosan-alginate scaffold is reported to increase distribution of cells inside the scaffold [ 21 ]. The even distribution, unlike dense cellular clusters, increases cell development and proliferation [ 22 ]. In terms of mechanical characteristics, the value of storage modulus of chitosan-alginate scaffold was same as the values of the peripheral area of a native TMJ disc and lower than the values of the central region [ 21 ].

### Fibrin

Fibrin gels mimic the coagulation process, and forms a fibrin clot, which attaches to the tissue and provide a cell adhesion site [ 23 ]. Addition of fibrin gel to fabricate a hybrid chitosan/fibrin scaffold increases cell seeding efficiency, cell spreading, biocompatibility, production of GAG content, expression of collagen type 1 compared to chitosan scaffold [ 24 ]. Although without cells, no sign of repair is observed *in vivo* [ 24 ].

### Synthetic Biomaterials

Despite all felicitous properties of natural scaffolds, they offer limited control over structural properties such as porosity, fiber diameter, and so on [ 25 ]. Although synthetic scaffolds have overcome this issue, they usually require additional processes to provide biomimetic cell signaling [ 25 ].

### Polycaprolactone

Polycaprolactone (PCL) is a biocompatible synthetic polymer with sufficient mechanical properties [ 26 ]. The stiffness of a PCL scaffold is even rather higher than the native disc, which can facilitate placement of the scaffold [ 6 ].The size, density, and direction of PCL fibers in a scaffold are matters of importance in determining mechanical properties, as well as temperature during production [ 5 - 6 ]. Filament enlargement from 200 µm to 300 µm would almost double the compressive modulus, which is probably related to a decrease in size of the pores; however, it decreases the yield stress [ 26 ]. The PCL fibers with a size of 300 µm and twice parallel fibers to the alignment direction than the perpendicular ones with biomimetic fiber alignment (the alignment that resembles collagen fibers’ alignment in peripheral and intermediate area), have shown a tensile modulus approximate to the TMJ disc’s [ 5 ]. Cross-arranged fibers of PCL endure forces from various directions and seem to be responsive to mechanical needs [ 27 ]. A PCL /PLA (polylactide) 2D nano-membrane in which fiber alignment evokes region-dependent collagen alignment in native disc, has shown a higher compressive modulus, relaxation modulus, and tensile strength compared to a membrane with random fiber alignment [ 28 ]. Direction of scaffold fibers not only determines mechanical properties but also influences the healing process and guides the arrangement of GAG content [ 28 ]. PCL/PLA nano-membrane with random fiber alignment could not completely repair a native TMJ disc that had gone through 80% subtotal discectomy after 16 weeks, whereas the same membrane with biomimetic fiber alignment could repair it thoroughly [ 28 ]. Temperature affects the mechanical properties by influencing porosity and scaffold’s geometry [ 6 ]. Higher temperature during fabrication, lessens the porosity, flattens the fibers, and enhances the possibility of change in the scaffolds structure [ 6 ]. A study suggested that a temperature rise from 80ºC to 86ºC decreases the compressive modulus about 8 MPa and [ 6 ]; however, another study suggested that an increase from 78ºC to 80ºC increases the compressive modulus and yield stress [ 26 ]. Since fibrocartilage regeneration time is rather long, slow degradation rate of a scaffold would benefit the regeneration process [ 29 ]. PCL polymer seems to have a long degradation time of 2 to 4 years, which is longer than desired [ 26 , 29 ]. Addition of poly lacticcoglycolic acid microspheres is believed to accelerate its degradation time to a more suitable time [ 30 ].

Connective tissue growth factor as a factor contributed to fibroblastic differentiation and formation of fibrous matrix and transforming growth factor- β3 (TGF- β3) as a factor contributed to chondrogenic differentiation and formation of cartilaginous matrix has been added to a stem cell- seeded PCL scaffold via poly lacticcoglycolic acid microspheres [ 5 ]. The former was added to the peripheral area and the latter to the intermediate area, in attempt to mimic native TMJ disc’s ECM matrix [ 5 ]. Addition of connective tissue growth factor and TGF- β3 not only resulted in a denser collagen type 1 matrix but also induced collage type 2 formation in the intermediate zone, whereas no collagen type 2 was found in their absence [ 5 , 30 ]. Besides, it increased the coefficient of viscosity in both intermediate and peripheral areas [ 5 ]. It turned out that higher concentration of mentioned growth factors could cause a denser cartilaginous matrix (collagen type 2 and aggrecan) and a lower compressive modulus (closer to that of native disc). The former consequently alters viscoelastic properties of the scaffold [ 31 ], enhances coefficient of viscosity and diminishes ratio of relaxation modulus to instantaneous modulus (make them closer to that of native disc) [ 5 ]. The mentioned scaffold presented a constant release of connective tissue growth factor and TGF-β3 up to 42 days [ 5 ]. In another study, addition of bone morphogenic protein-2 as a factor contributed to mineralization and collagen formation along with connective tissue growth factor and TGF- β3 to a stem cell- seeded PCL scaffold was investigated [ 30 ]. It presented a constant release of the factors up to 42 days, complete *in vivo* degradation and tissue replacement after 4 weeks, region- dependent ECM matrix same as native disc and collagen rich matrix whose fibers’ alignments were same as native disc [ 30 ].

### Poly (ethylene glycol) diacrylate

Application of hybrid constructs such as poly (ethylene glycol) diacrylate (PEGDA) hydrogels along with PCL scaffolds are believed to have great potential [ 32 ]. The reasoning is that PCL scaffold can provide proper mechanical properties, while the hydrogel filling the pores can provide proper cell and growth factor distribution and attachment [ 6 , 32 ]. Attempts have been made to improve mechanical properties of PEGDA hydrogel in combination with PCL scaffold. Increase in PEGDA concentration in hydrogel from 20% to 30%, doubles the compressive modulus and triples the ultimate strength; however, the hydrogel with 30% concentration has turned out to be highly brittle [ 6 ]. Increase in concentration also reduces the degradation rate [ 26 ]. Addition of PEGDA hydrogel in form of a core and a shell to a PCL scaffold has been studied [ 26 ]. The hydrogel core keeps the integrity of the structure, withstands large forces for a long period of time, and mimics mechanical properties of native disc; however, the hydrogel shell mimics the surface properties of native such as storing and diffusing synovial fluid, reducing friction, and reducing stress concentration by spreading forces [ 26 ].

The natural hydrophobicity of PCL scaffold have been tried to be diminished by NaOH solution surface treatment [ 6 ]. Although, NaOH solution improves hydrophilicity and thus cellular attachment, it decreases mechanical properties of PCL scaffold [ 6 ].

Carbon nanotubes are promising reinforcements due to mechanical strength and chemical stability [ 33 ]. Addition of carbon nanotubes to a PCL/PLA Nano-membrane up to 0.25 wt% could increase the tensile strength. However, region-dependent fiber alignment has a greater impact on compressive and relaxation modulus than addition of 0.25 wt% carbon nanotubes, and application of both has a synergistic effect [ 28 ]. Carbon nanotubes could also increase hydrophilicity, biocompatibility, cell distribution, cell proliferation, and fibro-chondrogenic differentiation [ 34 , 35 ]. A PCL/PLA nano-membrane with biomimetic fiber alignment and 0.25 wt% carbon nanotubes is capable of regenerating an anisotropic tissue with well-organized collagen fibers and GAG distribution same as native disc which majorly accumulates in the intermediate area and displays limited expression in the peripheral area [ 28 ].

PLA is a synthetic polymer with rather long degradation time, which makes it an option for tissue regeneration [ 36 ]. Addition of transforming growth factor β1 to a PLA scaffold seeded with adipose stem cells increase production of aggrecan and collagen type 1; however, the scaffold completely repairs a TMJ with 90% subtotal discectomy after 6 months whether with or without transforming growth factor β1 [ 36 ]. Although, the addition of transforming growth factor β1 results in a smoother tissue, which is more calcified near the condylar bone [ 36 ].

### Polyvinyl alcohol

Polyvinyl alcohol (PVA) is a hydrophilic synthetic polymer [ 37 ]. PVA in hydrogel form can provide lubrication by maintaining a substantial amount of water; however, it lacks suitable mechanical strength [ 27 , 38 ]. Recently, in an attempt to improve mechanical properties, addition of PCL implants into the PVA hydrogel has been studied [ 27 ]. PCL implants increase Young’s modulus of PVA hydrogel. PVA hydrogel+ PCL implant also illustrates sufficient tensile strength to withstand functional forces, less creep than native disc, proper viscoelastic properties, and it recovers initial maximum strength after cyclic compressive stress even better than the native TMJ disc of a goat, which means that it could absorb destructive energy [ 27 ]. Another problem with PVA, as well as PVA hydrogel+ PCL implant is that although they are biocompatible, their surface is not favorable for cell adhesion and proliferation; however, this helps them maintain surface smoothness and helps the disc’s movement [ 27 ].

### Poly (sodium styrene sulfonate) and Poly (dimethyl diallyl ammonium chloride)

Poly (sodium styrene sulfonate) and poly (dimethyl diallyl ammonium chloride) are two synthetic polymers. The effects of addition of TiO2 nanofilms, as a super-hydrophilic surface modification to a scaffold fabricated from the aforementioned polymers, have been inspected [ 4 , 39 ]. An increase in thickness of TiO2 nanolayer was contributed to enhanced cell proliferation and protein synthesis, and same as the native TMJ disc collagen type 1 surpassed the type 2 [ 4 ]. The scaffold also proved to be biocompatible and cell adherent [ 4 ]. 

### Poly (glycerol sebacate)

Poly (glycerol sebacate) is a biocompatible and biodegradable polymer [ 40 ]. Poly (glycerol sebacate) scaffold along with fibro-chondrocytes has shown random distribution of collagen and GAG. Higher fibro-chondrocytes seeding density results in higher cellular content, and EMC production [ 40 ]. Higher fibro-chondrocytes seeding density also results in a higher collagen and GAG content after 4 weeks [ 40 ]. 

### Decellularized tissues

Decellularized ECM scaffolds are considered cost-effective method for tissue regeneration, since they can maintain bioactive molecules such as growth factors [ 41 ]. Decellularized scaffolds derived from native TMJ disc are believed to be biocompatible, bioactive and maintain chemical and mechanical properties of the tissues they were derived from [ 1 , 42 - 43 ]. It is believed that decellularization changes the ECM architecture with compacting ECM fiber, which consequently increases the compressive modulus to an extreme extent, decreases permeability, and causes a non-uniform cell distribution [ 1 , 10 ]. There is also evidence of increased collagen and decreased GAG content [ 1 ]. CO2 laser is suggested to be helpful for managing the undesired alterations by producing organized microporosity in the scaffold, which improves permeability, correction of fibril structures, which results in a compressive modulus close to that of native disc and finally uniform cell distribution, which is probably attributed to the increase in elastic modulus [ 10 ]. The last one helps the scaffold degradation to be post-pones [ 10 ]. There is a debate that presence of abundant dense fibrils in a decellularized scaffold might limit cell proliferation [ 44 ]. Previous studies suggest that preparation of decellularized tissue in form of hydrogel overcome this challenge due to loose structure and fast degradation [ 1 , 10 ]. The downside is that fast degradation may not provide proper stability during the whole regeneration process [ 45 ]. The condition of decellularized hydrogel preparation can affect its mechanical properties [ 1 ]. It is argued that an increase in shear rate, temperature, and concentration of decellularized ECM decreases viscosity, increases storage modulus and increases compressive strength, respectively [ 1 ]. 

The greatest challenge right now might be finding an appropriate surgical approach for implementation of the scaffolds. Besides, proper clinical guidelines should be developed. We have come a long way but there is still a lot to do before tissue engineering becomes a routine clinical option. 

This narrative review preformed a search with both strengths and limitations. The use of two major databases, PubMed and Google Scholar, increased the probability of finding relevant studies. However, the search only included studies published within the last 10 years. Furthermore, the inclusion of only specific types of studies may limit the range of evidence. 

## Conclusion

Scaffolds are an essential part of TMJ disc regeneration. Natural biomaterials, synthetic biomaterials, and decellularized tissues are commonly used as scaffolds for this purpose. Although synthetic scaffolds offer more control over structural properties, natural scaffolds, such as collagen, hyaluronic acid, chitosan and alginate are still widely used due to their ability to promote cell attachment, signaling, and proliferation. The type of scaffold significantly affects the mechanical, structural, and biological properties of the regenerated tissue. Therefore, the development of an ideal scaffold that mimics the native TMJ disc is of great importance in the field of tissue engineering.

Although there is not a commercial product on the market yet, several *in vivo* studies have managed to prepare appropriate scaffolds for regeneration of TMJ disc. However, longitudinal studies are required to observe and evaluate the effect of these scaffolds in a long run. 
